# Supplementary material for: Comparative Chloroplast Genomics of Actinidia deliciosa Cultivars: Insights into Positive Selection and Population Evolution
Source: Int J Mol Sci. 2025 May 5;26(9):4387. doi: 10.3390/ijms26094387 (PMC12072308; doi:10.3390/ijms26094387)
Supplement: Supplementary file 1 [file ijms-26-04387-s001.zip › Figure S1-S3.pdf]

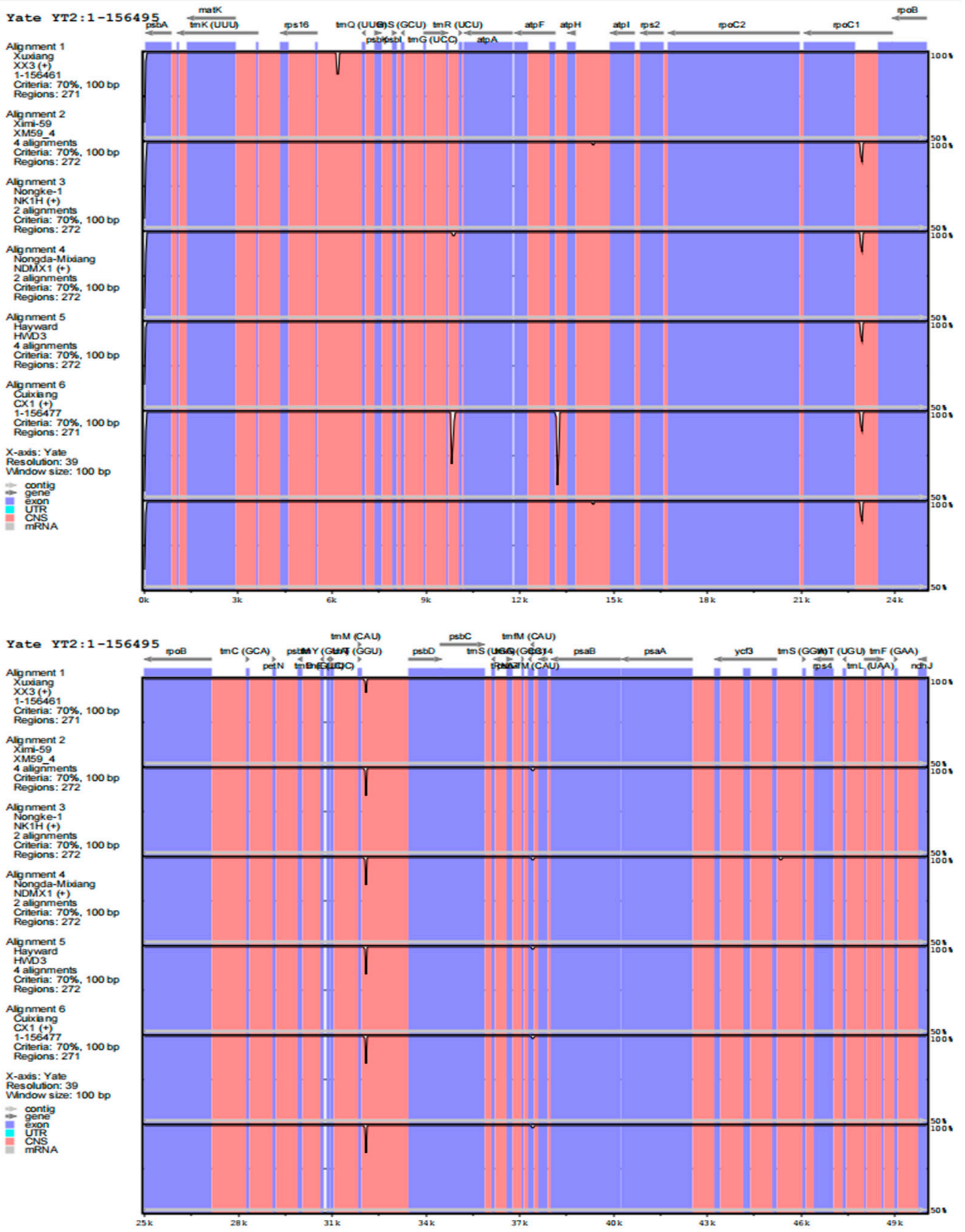

Yate YT2:1-156495
Alignment 1
Xuxiang
XX3 (+)
1-156461
Criteria: 70%, 100 bp
Regions: 271
Alignment 2
Ximi-59
XM59_4
4 alignments
Criteria: 70%, 100 bp
Regions: 272
Alignment 3
Nongke-1
NK1H (+)
2 alignments
Criteria: 70%, 100 bp
Regions: 272
Alignment 4
Nongda-Mixiang
NDMX1 (+)
2 alignments
Criteria: 70%, 100 bp
Regions: 272
Alignment 5
Hayward
HWD3
4 alignments
Criteria: 70%, 100 bp
Regions: 272
Alignment 6
Cuixiang
CX1 (+)
1-156477
Criteria: 70%, 100 bp
Regions: 271
X-axis: Yate
Resolution: 39
Window size: 100 bp
contig
gene
exon
UTR
CNS
mRNA
psbA
matK
trnK (UUU)
rps16
atpA
atpF
atpH
atpI
rps2
rpoC2
rpoC1
rpoB
100%
50%
0k
3k
6k
9k
12k
15k
18k
21k
24k
Yate YT2:1-156495
rpoB
trnC (GCA)
petN
trnM (CAU)
psbD
psbC
trnfM (CAU)
psaB
psaA
ycf3
rps4
trnL (UAA)
trnF (GAA)
ndhJ
25k
28k
31k
34k
37k
40k
43k
46k
49k

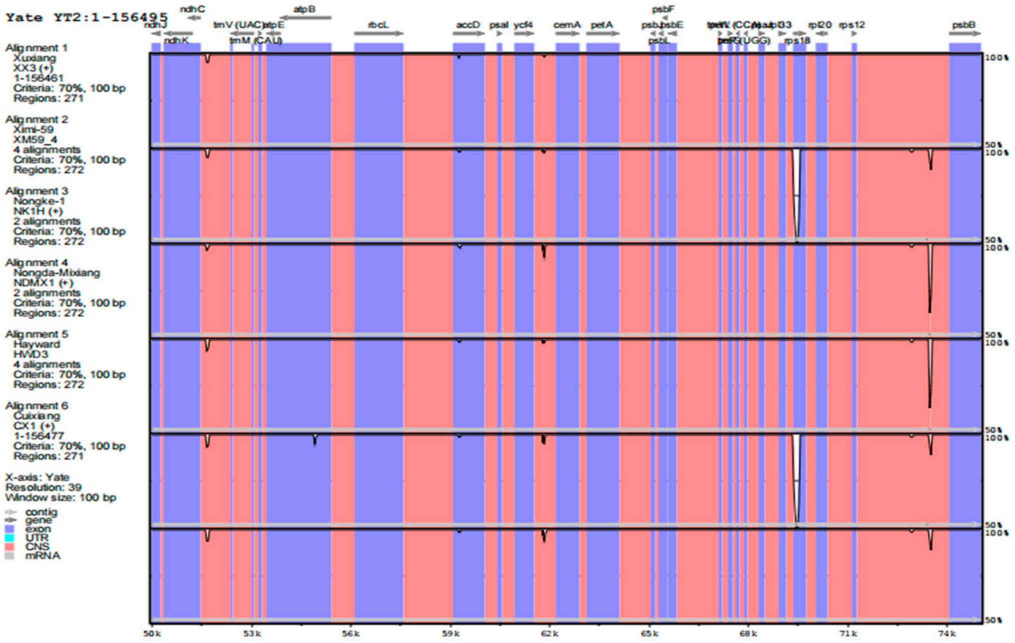

Yate YT2:1-156495
ndhJ
ndhC
ndhK
trnV (UAC)
trnM (CAU)
atpE
atpB
rbcL
accD
psaI
ycf4
cemA
petA
psbF
psbE
psbL
rpl33
rps18
rpl20
rps12
psbB
Alignment 1
Xuxiang
XX3 (+)
1-156461
Criteria: 70%, 100 bp
Regions: 271
Alignment 2
Ximi-59
XM59_4
4 alignments
Criteria: 70%, 100 bp
Regions: 272
Alignment 3
Nongke-1
NK1H (+)
2 alignments
Criteria: 70%, 100 bp
Regions: 272
Alignment 4
Nongda-Mixiang
NDMX1 (+)
2 alignments
Criteria: 70%, 100 bp
Regions: 272
Alignment 5
Hayward
HWD3
4 alignments
Criteria: 70%, 100 bp
Regions: 272
Alignment 6
Cuixiang
CX1 (+)
1-156477
Criteria: 70%, 100 bp
Regions: 271
X-axis: Yate
Resolution: 39
Window size: 100 bp
contig
gene
exon
UTR
CNS
mRNA
100%
50%
50k
53k
56k
59k
62k
65k
68k
71k
74k

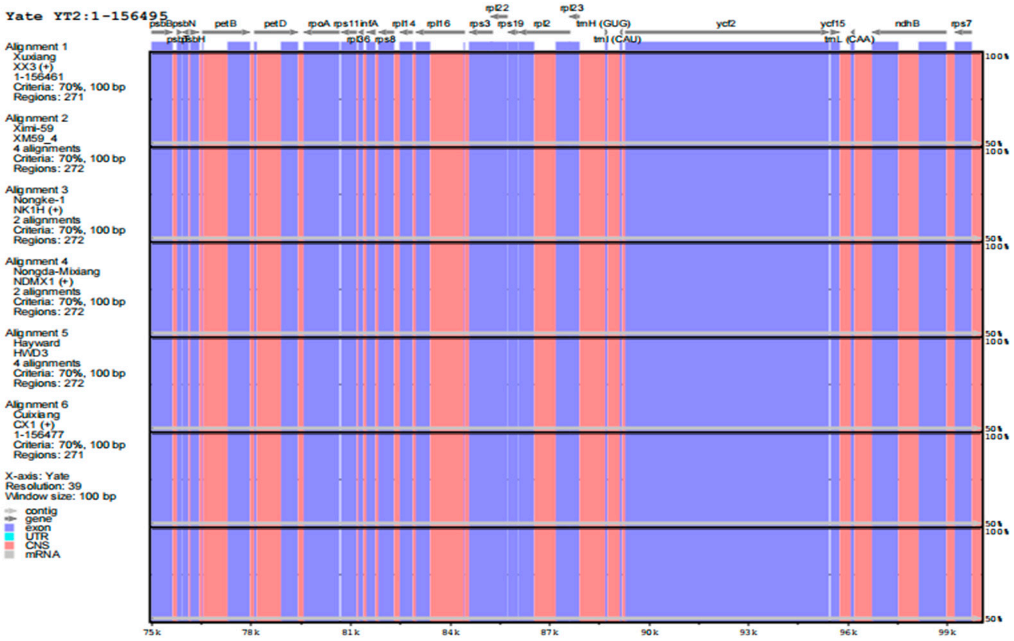

Yate YT2:1-156495
psbN
psbH
petB
petD
rpoA
rps11
infA
rpl36
rps8
rpl14
rpl16
rps3
rpl22
rps19
rpl2
rpl23
trnH (GUG)
trnI (CAU)
ycf2
ycf15
trnL (CAA)
ndhB
rps7
Alignment 1
Xuxiang
XX3 (+)
1-156461
Criteria: 70%, 100 bp
Regions: 271
Alignment 2
Ximi-59
XM59_4
4 alignments
Criteria: 70%, 100 bp
Regions: 272
Alignment 3
Nongke-1
NK1H (+)
2 alignments
Criteria: 70%, 100 bp
Regions: 272
Alignment 4
Nongda-Mixiang
NDMX1 (+)
2 alignments
Criteria: 70%, 100 bp
Regions: 272
Alignment 5
Hayward
HWD3
4 alignments
Criteria: 70%, 100 bp
Regions: 272
Alignment 6
Cuixiang
CX1 (+)
1-156477
Criteria: 70%, 100 bp
Regions: 271
X-axis: Yate
Resolution: 39
Window size: 100 bp
contig
gene
exon
UTR
CNS
mRNA
100%
50%
75k
78k
81k
84k
87k
90k
93k
96k
99k

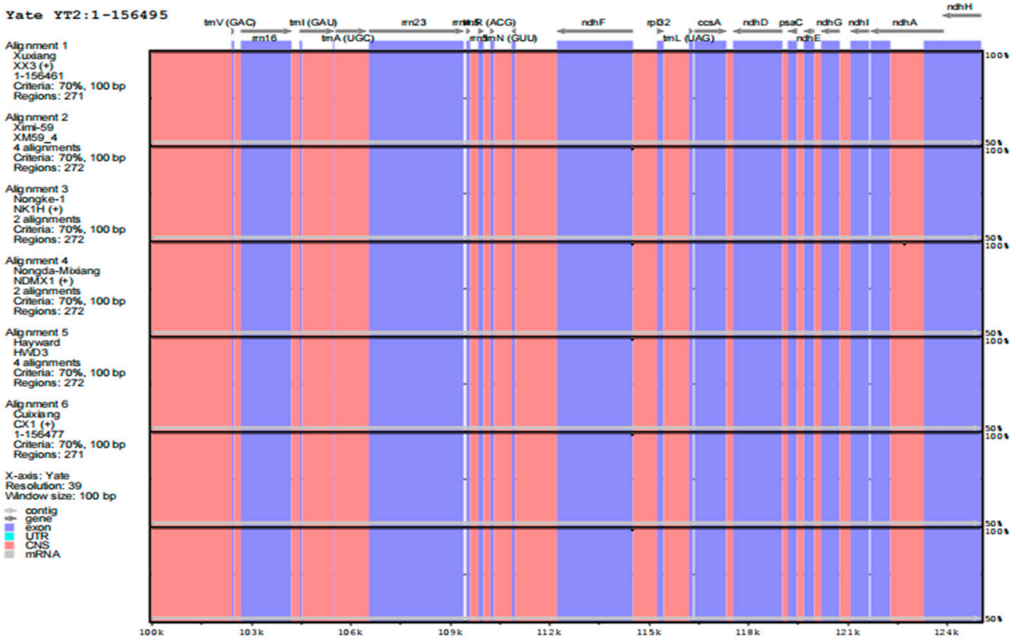
Yate YT2:1-156495
Alignment 1
Xuxiang
XX3 (+)
1-156461
Criteria: 70%, 100 bp
Regions: 271
Alignment 2
Ximi-59
XM59_4
4 alignments
Criteria: 70%, 100 bp
Regions: 272
Alignment 3
Nongke-1
NK1H (+)
2 alignments
Criteria: 70%, 100 bp
Regions: 272
Alignment 4
Nongda-Mixiang
NDMX1 (+)
2 alignments
Criteria: 70%, 100 bp
Regions: 272
Alignment 5
Hayward
HWD3
4 alignments
Criteria: 70%, 100 bp
Regions: 272
Alignment 6
Cuixiang
CX1 (+)
1-156477
Criteria: 70%, 100 bp
Regions: 271
X-axis: Yate
Resolution: 39
Window size: 100 bp
contig
gene
exon
UTR
CNS
mRNA
trnV (GAC)
rrn16
trnI (GAU)
trnA (UGC)
rrn23
trnR (ACG)
trnN (GUU)
ndhF
rpl32
trnL (UAG)
ccsA
ndhD
psaC
ndhE
ndhG
ndhI
ndhA
ndhH
100%
50%
100k
103k
106k
109k
112k
115k
118k
121k
124k

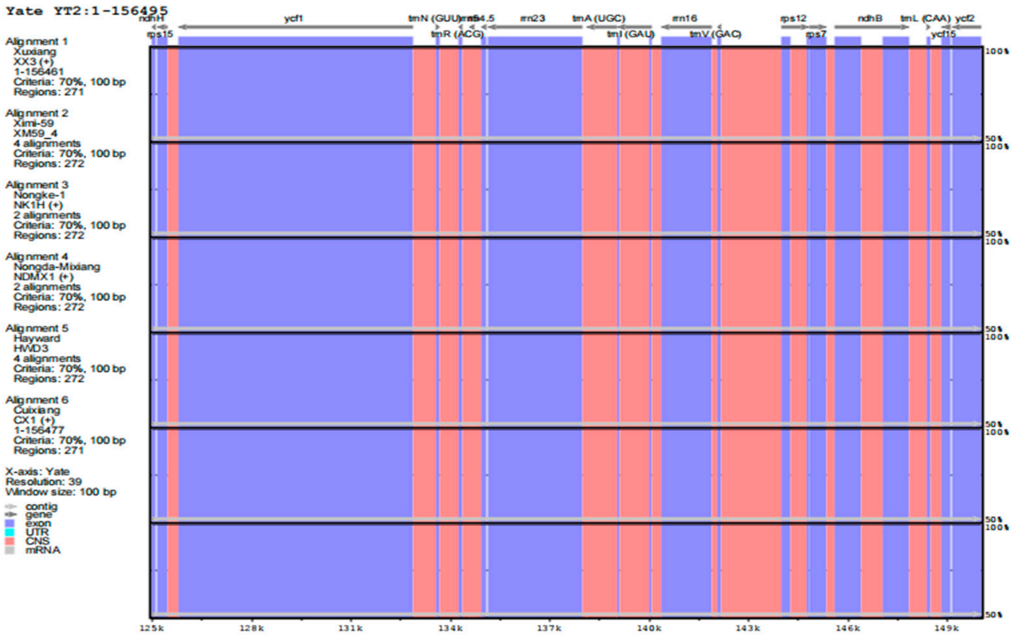
Yate YT2:1-156495
Alignment 1
Xuxiang
XX3 (+)
1-156461
Criteria: 70%, 100 bp
Regions: 271
Alignment 2
Ximi-59
XM59_4
4 alignments
Criteria: 70%, 100 bp
Regions: 272
Alignment 3
Nongke-1
NK1H (+)
2 alignments
Criteria: 70%, 100 bp
Regions: 272
Alignment 4
Nongda-Mixiang
NDMX1 (+)
2 alignments
Criteria: 70%, 100 bp
Regions: 272
Alignment 5
Hayward
HWD3
4 alignments
Criteria: 70%, 100 bp
Regions: 272
Alignment 6
Cuixiang
CX1 (+)
1-156477
Criteria: 70%, 100 bp
Regions: 271
X-axis: Yate
Resolution: 39
Window size: 100 bp
contig
gene
exon
UTR
CNS
mRNA
ndhH
rps15
ycf1
trnN (GUU)
trnR (ACG)
rrn23
trnA (UGC)
trnI (GAU)
rrn16
trnV (GAC)
rps12
rps7
ndhB
trnL (CAA)
ycf2
ycf15
100%
50%
125k
128k
131k
134k
137k
140k
143k
146k
149k

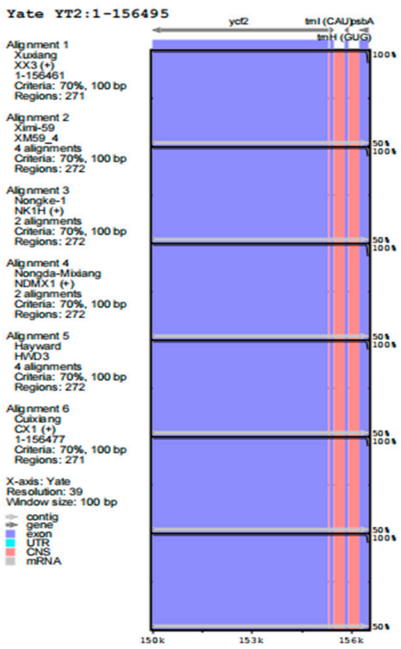

Figure S1. Sequence identity plots based on 7 *A. deliciosa* chloroplast genomes, with *A. deliciosa* cv. Yate as a reference. Sequences of chloroplast genomes were aligned and compared using the mVISTA program. Annotated genes are displayed along the top. The vertical scale indicates the percentage identity ranging from 50 to 100%. Genome regions are color coded as exon, conserved non-coding sequences (CNS), and mRNA.

a

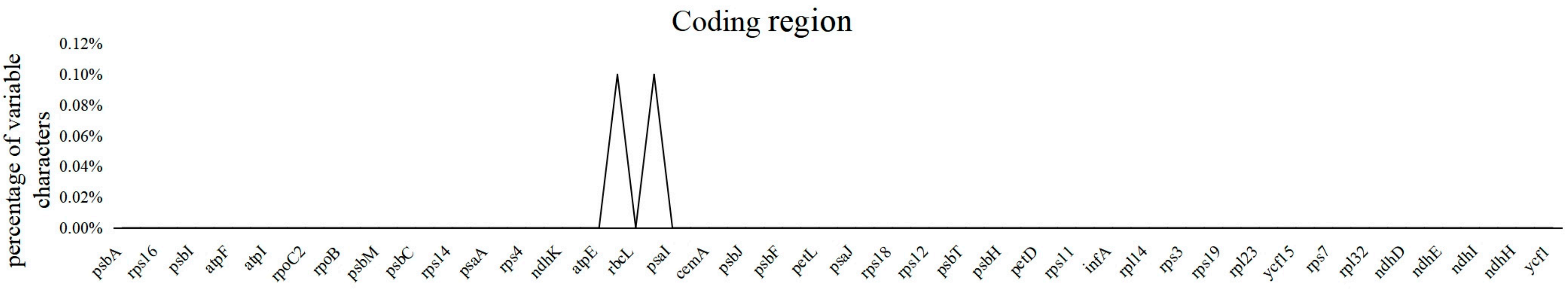

b

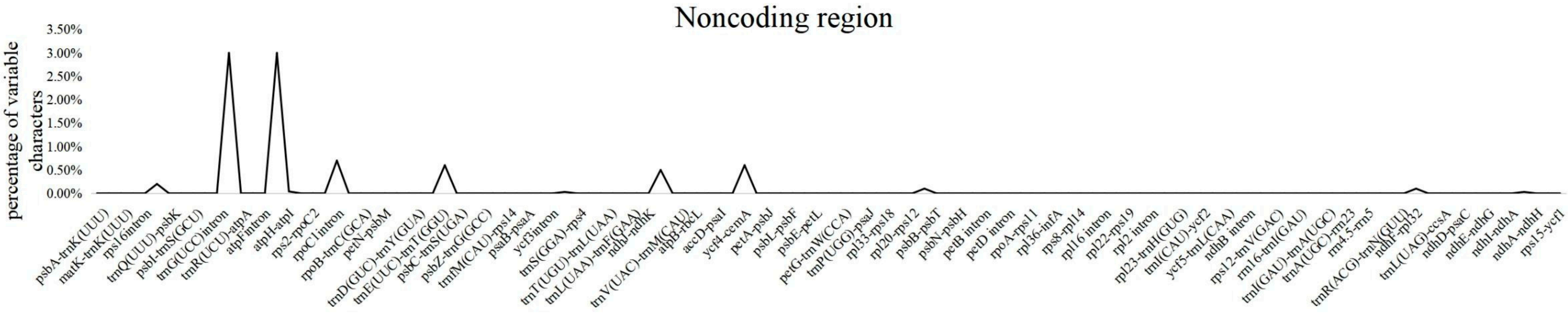

Figure S2. Percentages of variable characters in homologous regions among *A. deliciosa* chloroplast genomes. (a) Coding regions. (b) Non-coding regions. The homologous regions are oriented according to their locations in the chloroplast genome.

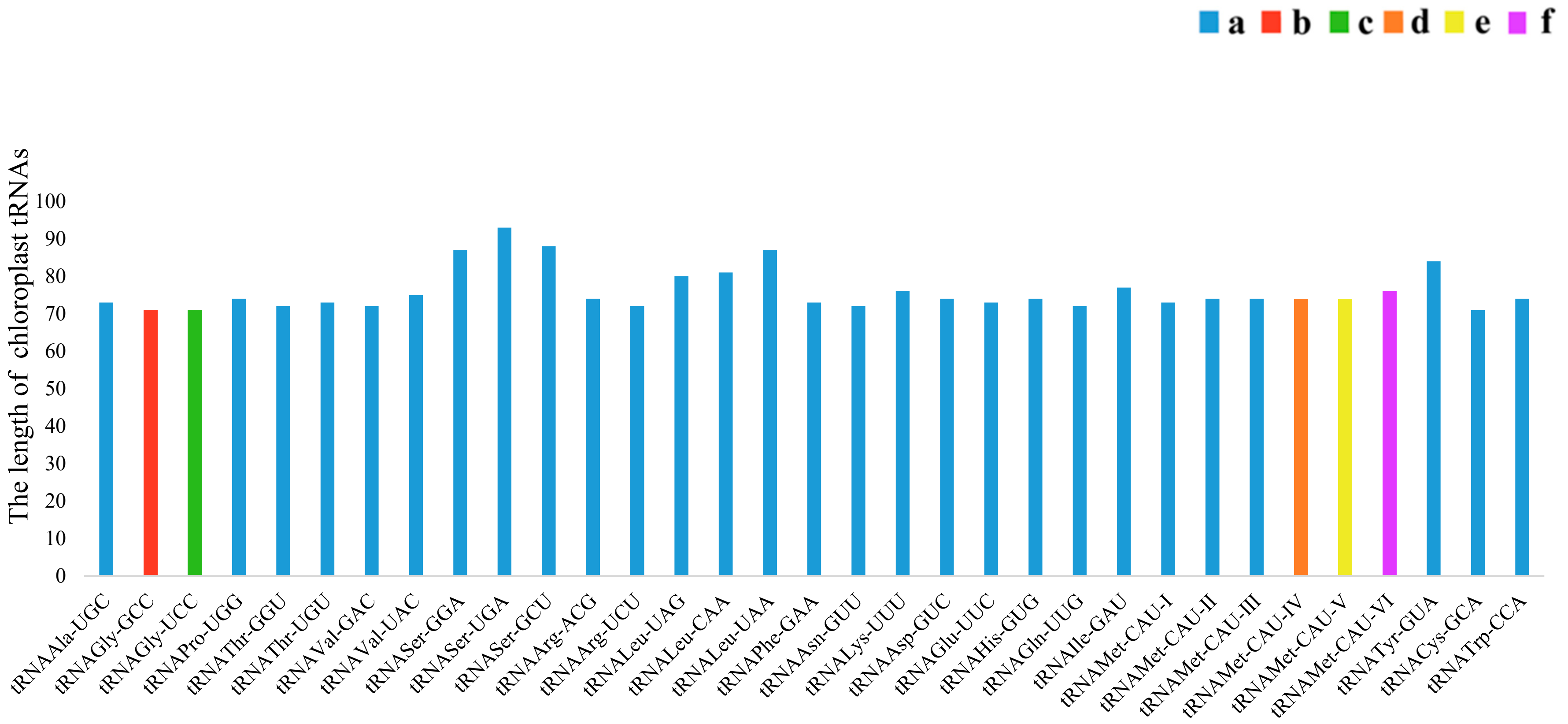

Figure S3. The length of *Actinidia* chloroplast tRNAs. a tRNA length of all the species; b $tRNA^{Gly}$-GCC length of all the species except *A. kolomikta*, *A. eriantha*, *A. callosa* var. *strigillosa*, *A. arguta*, *A. rufa*, *A. tetramera*, *A. lanceolata* and *A. zhejiangensis*; c $tRNA^{Gly}$-UCC length of all the species except *A. chinensis* var. *setosa*, *A. valvata*, *A. fulvicoma* and *A. callosa* var. *henryi*; d $tRNA^{Met}$-CAU-IV length of *A. kolomikta*, *A. eriantha*, *A. arguta* and *A. tetramera*; e $tRNA^{Met}$-CAU-V length of *A. latifolia*; f $tRNA^{Met}$-CAU-VI length of *A. latifolia*.
